# p75NTR optimizes the osteogenic potential of human periodontal ligament stem cells by up‐regulating α1 integrin expression

**DOI:** 10.1111/jcmm.15390

**Published:** 2020-05-18

**Authors:** Jun Li, Manzhu Zhao, Yingying Wang, Mengjie Shen, Shuai Wang, Mengying Tang, Meng Li, Yuting Luo, Kun Yang, Xiujie Wen

**Affiliations:** ^1^ Department of Stomatology Daping Hospital Army Medical University (Third Military Medical University) Chongqing China; ^2^ College of Stomatology Chongqing Medical University Chongqing China; ^3^ Hospital of Stomatology Zunyi Medical University Zunyi China; ^4^ Hospital of Stomatology Southwest Medical University Luzhou China

**Keywords:** cell surface marker, human periodontal ligament stem cells, osteogenic differentiation, regenerative medicine, signalling pathway

## Abstract

Human periodontal ligament stem cells (hPDLSCs) are a promising source in regenerative medicine. Due to the complexity and heterogeneity of hPDLSCs, it is critical to isolate homogeneous hPDLSCs with high regenerative potential. In this study, p75 neurotrophin receptor (p75NTR) was used to isolate p75NTR^+^ and p75NTR^−^ hPDLSCs by fluorescence‐activated cell sorting. Differences in osteogenic differentiation among p75NTR^+^, p75NTR^−^ and unsorted hPDLSCs were observed. Differential gene expression profiles between p75NTR^+^ and p75NTR^−^ hPDLSCs were analysed by RNA sequencing. α1 Integrin (ITGA1) small interfering RNA and ITGA1‐overexpressing adenovirus were used to transfect p75NTR^+^ and p75NTR^−^ hPDLSCs. The results showed that p75NTR^+^ hPDLSCs demonstrated superior osteogenic capacity than p75NTR^−^ and unsorted hPDLSCs. Differentially expressed genes between p75NTR^+^ and p75NTR^−^ hPDLSCs were highly involved in the extracellular matrix‐receptor interaction signalling pathway, and p75NTR^+^ hPDLSCs expressed higher ITGA1 levels than p75NTR^−^ hPDLSCs. ITGA1 silencing inhibited the osteogenic differentiation of p75NTR^+^ hPDLSCs, while ITGA1 overexpression enhanced the osteogenic differentiation of p75NTR^−^ hPDLSCs**.** These findings indicate that p75NTR optimizes the osteogenic potential of hPDLSCs by up‐regulating ITGA1 expression, suggesting that p75NTR can be used as a novel cell surface marker to identify and purify hPDLSCs to promote their applications in regenerative medicine.

**Abbreviations**
ALP: alkaline phosphatase
CNC: cranial neural crest
ECM: extracellular matrix
EMSCs: ectomesenchymal stem cells
FBS: foetal bovine serum
hPDLSCs: human periodontal ligament stem cells
ITGA1: α1 integrin
ITGA7: α7 integrin
ITGA8: α8 integrin
NC: negative control
p75NTR: p75 neurotrophin receptor
qPCR: quantitative real‐time polymerase chain reaction
RUNX2: RUNX family transcription factor 2
MSCs: mesenchymal stem cells
siRNA: small interfering RNA

## 1 INTRODUCTION

Mesenchymal stem cells (MSCs) derived from teeth represent a fascinating area of research in regenerative medicine due to the complex and unique developmental origin of teeth.^1^ Human periodontal ligament stem cells (hPDLSCs) are odontogenic MSCs that were first identified in and isolated from periodontal ligament tissue by Seo et al in 2004.^2^ hPDLSCs were reported to have the ability to self‐renew and the potential to differentiate into various specialized cell types, such as osteoblasts, fibroblasts, cementoblasts, chondroblasts, adipoblasts and neuroblasts.^2^, ^3^, ^4^, ^5^, ^6^ Further evidence showed that hPDLSCs had little immunogenicity, maintained a higher growth capacity and were easy to obtain from impacted third molars or premolars extracted for orthodontics, demonstrating a promising application in regenerative medicine.^5^, ^6^, ^7^, ^8^ Therefore, hPDLSCs have been widely investigated in recent studies. However, as a complex and heterogeneous population, hPDLSCs even within the same condition may reflect different biological properties, restricting their utilization as stem cells.^1^, ^9^ Further isolation of homogeneous hPDLSCs with high regenerative potential will greatly contribute to their applications in the future.

During tooth development, ectomesenchymal stem cells (EMSCs) originating from the cranial neural crest (CNC) differentiate into various mesenchymal cell lines, such as dental papilla cells, pre‐odontoblasts and dental follicle cells, ultimately giving rise to pulp, dentine, cementum and periodontal ligaments.^10^, ^11^ Some scholars have suggested that a population of the CNC‐derived progenitor cells remain in the periodontal ligaments and continue to maintain their differentiation potential, namely periodontal ligament stem cells.^12^ More accurate isolation of these CNC‐derived stem cells will promote their use in the studies of differentiation mechanisms and in tissue engineering applications. p75 neurotrophin receptor (p75NTR) is a well‐conserved transmembrane neurotrophin/proneurotrophin receptor that belongs to the tumour necrosis factor receptor superfamily.^13^, ^14^ The role of p75NTR is not limited to the nervous system, as it may have multifarious biological functions in non‐neuronal tissues during development and differentiation.^15^, ^16^ In addition, p75NTR is highly expressed in CNC‐derived cells and proved to be a reliable cell surface marker for stem cells from the CNC.^17^ In our previous study, p75NTR was successfully used as a cell surface marker to isolate p75NTR^+^ EMSCs from rats.^18^, ^19^ Further research found that these p75NTR^+^ EMSCs, as CNC‐derived stem cells, displayed superior multi‐lineage differentiation potential and that p75NTR was involved in the positive regulation of osteogenic differentiation of rat EMSCs.^20^, ^21^ However, few studies that isolate hPDLSCs based on p75NTR expression have been reported, and the effect and underlying mechanism of p75NTR on the osteogenic potential of hPDLSCs are also unclear.

The present study aimed to use p75NTR as a cell surface marker to isolate hPDLSCs and to further investigate the effect and underlying mechanism of p75NTR on the osteogenic potential of hPDLSCs. We expected to find an optimized cell surface marker for the identification and purification of hPDLSCs, thus promoting their applications in regenerative medicine.

## 2 MATERIALS AND METHODS

### 2.1 Cell isolation and culture

Tissues were obtained under approved guidelines set by Zunyi Medical University with informed patient consent. Primary hPDLSCs were isolated from premolars extracted from 18‐ to 25‐year‐old patients undergoing orthodontic treatment. Briefly, the periodontal ligaments were dissected from the root surface and digested with 1% collagenase I at 37°C for 45 minutes and neutralized with minimum essential medium α (α‐MEM; Gibco, Waltham, MA, USA) containing 10% foetal bovine serum (FBS; Gibco). Then, the cell suspension was centrifuged at 800 rpm for 5 minutes. Next, the cell pellet was resuspended in α‐MEM supplemented with 10% FBS and antibiotics (100 μg/mL streptomycin and 100 μg/mL penicillin) and then cultured at 37°C in a 5% CO2 humidified incubator.

### 2.2 Fluorescence‐activated cell sorting

Third‐passage hPDLSCs were digested with a 1% trypsin/1 mM EDTA solution (Solarbio, Beijing, China) and suspended in 3 mL phosphate‐buffered saline with 3% FBS. Then, the specimens were incubated with mouse anti‐human p75NTR (1:20; BD Pharmingen™, Franklin Lakes, NJ, USA) at 37°C for 60 minutes. Next, p75NTR^+^ and p75NTR^−^ hPDLSCs were sorted by flow cytometry (MoFlo XDP; Beckman Coulter, Brea, CA, USA) using mouse anti‐human IgG1 (1:20; BD Pharmingen™) as an isotype control.

### 2.3 Flow cytometry analysis

Fourth‐passage cells (5 × 10^5^) of each group were harvested and fixed with 4% paraformaldehyde for 30 minutes, followed by incubation with mouse anti‐human p75NTR, mouse anti‐human CD44, mouse anti‐human CD73, mouse anti‐human CD90, mouse anti‐human CD105, mouse anti‐human CD11b, mouse anti‐human CD19, mouse anti‐human CD34, mouse anti‐human CD45 and mouse anti‐human HLA‐DR antibodies (1:20; BD Pharmingen™) at 4°C for 2 hours. Subsequently, the specimens were analysed by flow cytometry.

### 2.4 Immunofluorescence assays

Fourth‐passage cells (2 × 10^5^) of each group were harvested and seeded onto confocal dishes for 12 hours. Then, the specimens were fixed with 4% polyoxymethylene for 30 minutes and incubated with rabbit anti‐human p75NTR (1:200; Abcam, Cambridge, MA, USA) or rabbit anti‐human α1 Integrin (ITGA1) (1:200; Abcam) at 4°C for 12 hours. To detect primary antibodies, goat anti‐rabbit IgG‐TRITC (Beyotime, Shanghai, China) secondary antibody was added and incubated for 1 hour at 37°C. Subsequently, the specimens were counterstained with DAPI (Solarbio) and observed under a confocal laser scanning microscope (TCS SP2; Leica Microsystems, Heidelberg, Germany).

### 2.5 Induction of adipogenic, chondrogenic and osteogenic differentiation

Fourth‐passage cells (2 × 10^5^) of each group were harvested and seeded onto 6‐well plates for 12 hours. Then, the specimens were cultured with adipogenic (α‐MEM supplemented with 5% FBS, 100 μg/mL streptomycin, 100 μg/mL penicillin, 1 μmol/L dexamethasone, 0.2 mmol/L indomethacin, 10 μg/mL insulin and 0.5 mmol/L IBMX), chondrogenic (α‐MEM supplemented with 5% FBS, 100 μg/mL streptomycin, 100 μg/mL penicillin, 0.1 μmol/L dexamethasone, 10 ng/mL TGFB1, 35 μg/mL ascorbic acid, 100 μg/mL sodium pyruvate, 40 μg/mL proline and 6.25 μg/mL insulin) or osteogenic (α‐MEM supplemented with 5% FBS, 100 μg/mL streptomycin, 100 μg/mL penicillin, 10 μmol/L dexamethasone, 50 μg/mL ascorbic acid and 10 mmol/L β‐glycerol phosphate disodium salt pentahydrate) induction medium. The medium was replaced every 3 days.

### 2.6 Oil Red O staining

The specimens were washed twice with PBS, fixed in 4% paraformaldehyde for 30 minutes, soaked with 60% isopropanol for 5 minutes and stained with an Oil Red O Stain Kit (Solarbio) for 20 minutes. After washing 3 times with double‐distilled water, the specimens were observed by optical microscope.

### 2.7 Alcian blue staining

The specimens were washed twice with PBS, fixed in 4% paraformaldehyde for 30 minutes and stained with an Alcian Blue Stain Kit (Solarbio) for 40 minutes. After washing three times with double‐distilled water, the specimens were observed by optical microscope.

### 2.8 Alkaline phosphatase staining

The specimens were washed twice with PBS, fixed in 4% paraformaldehyde for 30 minutes and stained with an Alkaline Phosphatase Activity Kit (Beyotime) for 30 minutes. After washing three times with double‐distilled water, the specimens were observed by optical microscope.

### 2.9 Alizarin Red staining

The specimens were washed twice with PBS, fixed in 4% paraformaldehyde for 30 minutes and stained with Alizarin Red S solution (Solarbio) for 10 minutes. After washing 3 times with double‐distilled water, the specimens were observed by optical microscope.

### 2.10 Quantitative real‐time polymerase chain reaction

Total RNA was extracted with RNAiso Plus (TaKaRa, Dalian, China). Reverse transcription of isolated RNA was performed by using PrimeScript™ RT Master Mix (TaKaRa). The concentrations of RNA and cDNA were detected by an ultraviolet spectrophotometer (NanoDrop Lite; Thermo Scientific, Waltham, MA, USA). Quantitative real‐time polymerase chain reaction (qPCR) was performed with TB Green™ Premix Ex Taq™ II (TaKaRa) and using GAPDH as a control (CFX Connect; Bio‐Rad, Hercules, CA, USA). Three independent samples from each group were used in the analysis (n = 3). The relative gene expression level was calculated by using the comparative Ct (2^−ΔΔCT^) method. The primer sets are listed in Table 1.

**Table 1:** Information of primer sequences

Gene	Primer sequence
p75NTR	Forward: CATCCCTGTCTATTGCTCCATC
	Reverse: GAGTTTTTCTCCCTCTGGTGG
ALP	Forward: CTGGTACTCAGACAACGAGATG
	Reverse: GTCAATGTCCCTGATGTTATGC
RUNX2	Forward: AGGCAGTTCCCAAGCATTTCATCC
	Reverse: TGGCAGGTAGGTGTGGTAGTGAG
ITGA1	Forward: AAGTGACCATGAATTTTGAGCC
	Reverse: CACATCAAAACACACTGTAGCA
ITGA7	Forward: TTGAGCTGCCACTGTCCATTGC
	Reverse: CCGTGACCTCATACTTGACCTTGC
ITGA8	Forward: GATTATGTTGGAATCGAACGCA
	Reverse: TTGTTTTCTCAAGACGTGGAAC
GAPDH	Forward: GTGGACCTGACCTGCCGTCTAG
	Reverse: GTGTCGCTGTTGAAGTCAGAGGAG

### 2.11 Western blot analysis

Western blot was conducted as described previously.^19^ The primary antibodies were rabbit anti‐human p75NTR (1:1000; Abcam), rabbit anti‐human alkaline phosphatase (ALP) (1:500; Abcam), rabbit anti‐human RUNX family transcription factor 2 (RUNX2) (1:1000; Abcam) and rabbit anti‐human α1 integrin (ITGA1) (1:500; Abcam). Rabbit anti‐human GAPDH (1:10 000; Proteintech, Wuhan, China) was used as an internal standard.

### 2.12 RNA sequencing and bioinformatics analysis

Total RNA was extracted from the cells with RNAiso Plus (TaKaRa). Any contaminating deoxyribonucleic acids were removed by DNase I digestion. Three independent cell samples from each group were used in the analysis (n = 3). After cDNA libraries were constructed, Illumina sequencing was performed on the libraries by Novogene Corporation (Beijing, China). The clean reads were obtained through raw data filtering, sequencing error rate examination and GC content distribution examination and were then compared to the reference genome using HISAT2 software. Next, the gene expression level was quantified by using featureCounts software. Subsequently, statistical analysis was performed on the expression data, and |log2(FoldChange)|>0 and *P*‐adj < 0.05 were used as the criteria to identify the differentially expressed genes. Then, clusterProfiler software was used to carry out KEGG pathway enrichment analysis.

### 2.13 Transfection of small interfering RNA

Fourth‐passage cells (2 × 10^5^) of each group were harvested and seeded onto 6‐well plates for 12 hours. Then, the specimens were transfected with ITGA1 small interfering RNA (siRNA) or negative control (NC) siRNA (100 pmol/well; Sangon, Shanghai, China) using Lipofectamine 2000 (1:50; Invitrogen, Carlsbad, CA, USA) for 6 hours.

### 2.14 Transfection of adenovirus

Fourth‐passage cells (2 × 10^5^) of each group were harvested and seeded onto 6‐well plates for 12 hours. Then, the specimens were transfected with ITGA1‐overexpressing adenovirus or NC‐overexpressing adenovirus (MOI = 30; Sangon) using polybrene (5 μg/ml; Hanbio, Shanghai, China) for 4 hours.

### 2.15 Statistical analysis

All data are expressed as the mean ± standard deviation. Statistical significance was assessed by using Student's *t* test for two groups or one‐way analysis of variance for three or more groups. *P* < 0.05 was considered as statistically significant.

## 3 RESULTS

### 3.1 p75NTR^+^, p75NTR^−^ and unsorted hPDLSC characteristics

**Figure 1:**
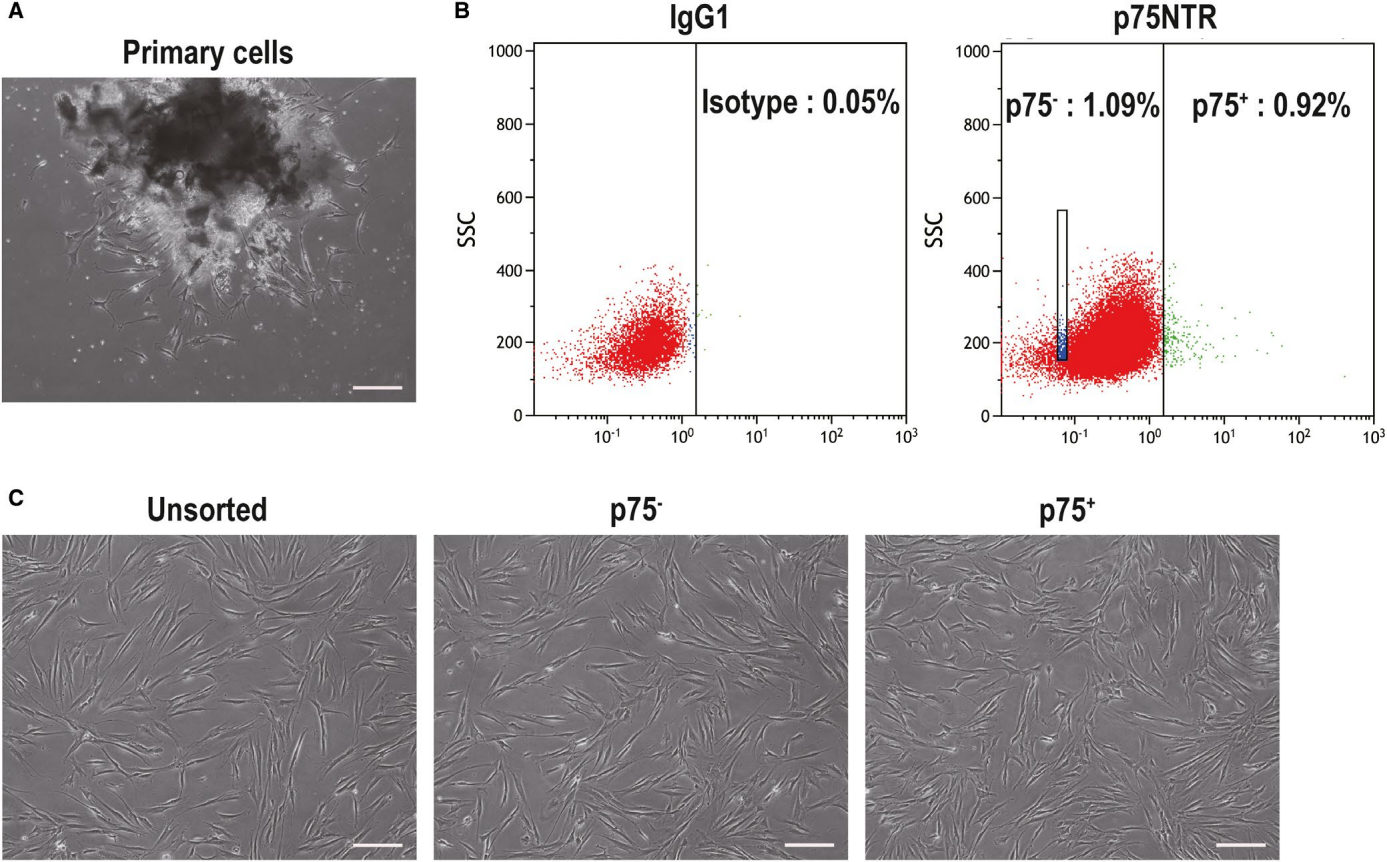
Sorting of p75NTR^+^ and p75NTR^−^ hPDLSCs from hPDLSCs isolated from periodontal ligament. (A) Primary hPDLSCs were observed by optical microscopy; scale bar = 100 μm. (B) Cells were sorted by fluorescence‐activated cell sorting. (C) The morphologies of p75NTR^+^, p75NTR^−^ and unsorted hPDLSCs were observed by optical microscopy; scale bar = 100 μm

**Figure 2:**
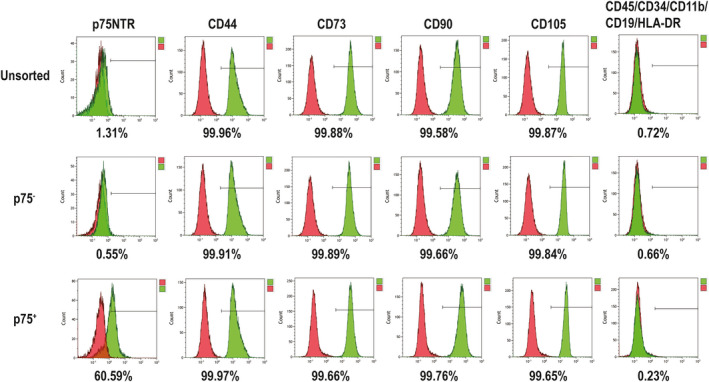
Flow cytometry analysis of the expression of cell surface markers. These cell surface markers related to p75NTR or mesenchymal (CD44, CD73, CD90 and CD105) or negative (CD45, CD34, CD11b, CD19 and HLA‐DR)

**Figure 3:**
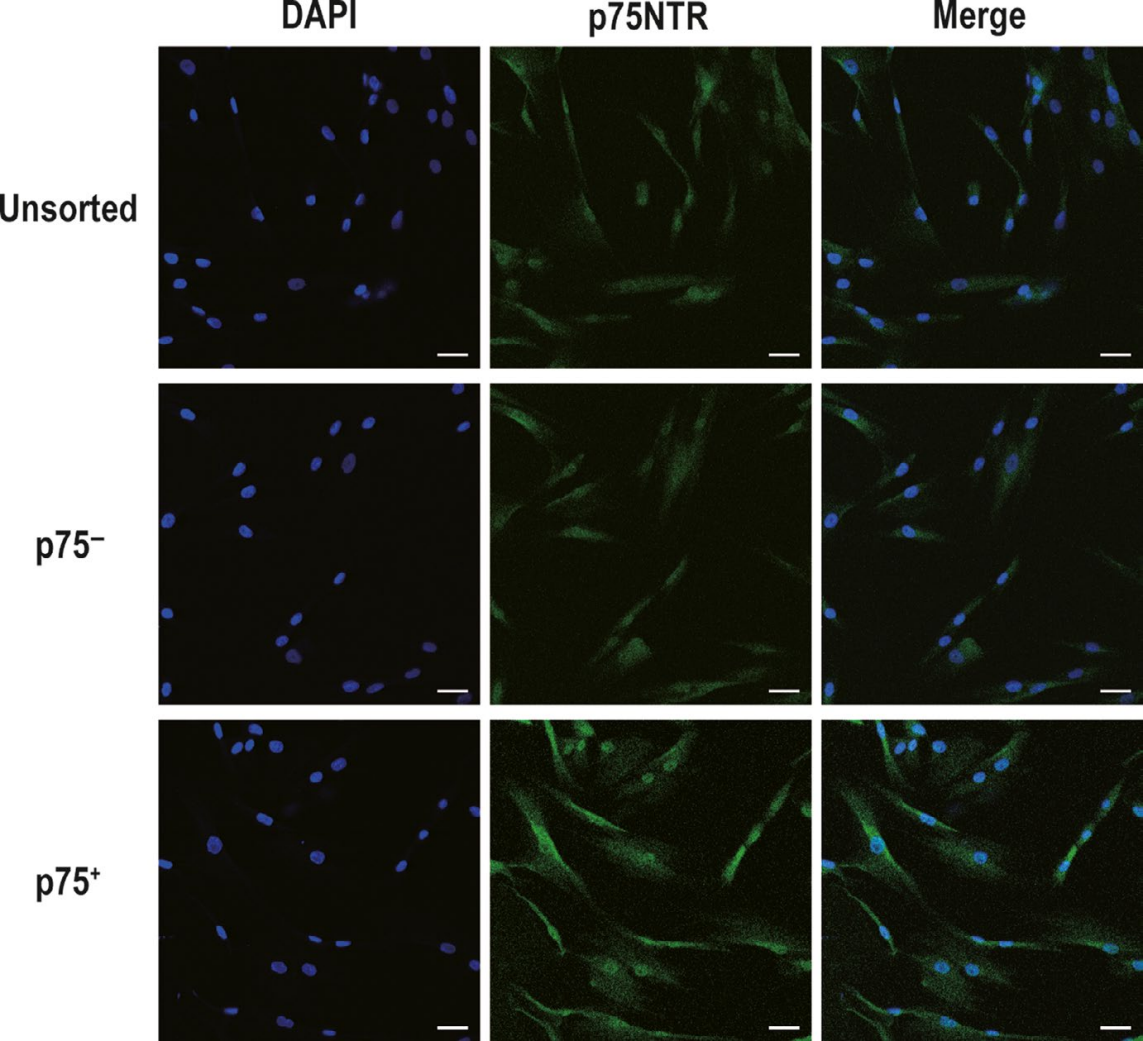
Confocal laser scanning microscopy results of the expression of p75NTR in p75NTR^+^, p75NTR^−^ and unsorted hPDLSCs; scale bar = 50 μm

Fluorescence‐activated cell sorting showed that p75NTR^+^ hPDLSCs accounted for 0.92% of the isolated hPDLSCs (Figure 1B). p75NTR^+^, p75NTR^−^ and unsorted hPDLSCs showed a long spindle morphology, which is morphologically characteristic of MSCs (Figure 1C). Moreover, flow cytometry analysis showed that MSC markers (CD44, CD73, CD90 and CD105) were highly expressed in p75NTR^+^, p75NTR^−^ and unsorted hPDLSCs, while MSC‐negative markers (CD45, CD34, CD11b, CD19 and HLA‐DR) were expressed at low levels in the three kinds of cells (Figure 2). Moreover, the expression rates of p75NTR were 60.59% in p75NTR^+^ hPDLSCs, 0.55% in p75NTR^−^ hPDLSCs and 1.31% in unsorted hPDLSCs (Figure 2). This result was consistent with the results of the confocal laser scanning microscopy assay, in which p75NTR was significantly enhanced in p75NTR^+^ hPDLSCs, while it was weakly expressed in p75NTR^−^ and unsorted hPDLSCs (Figure 3).

### 3.2 p75NTR^+^ hPDLSCs demonstrated superior osteogenic potential compared with p75NTR^−^ and unsorted hPDLSCs

**Figure 4:**
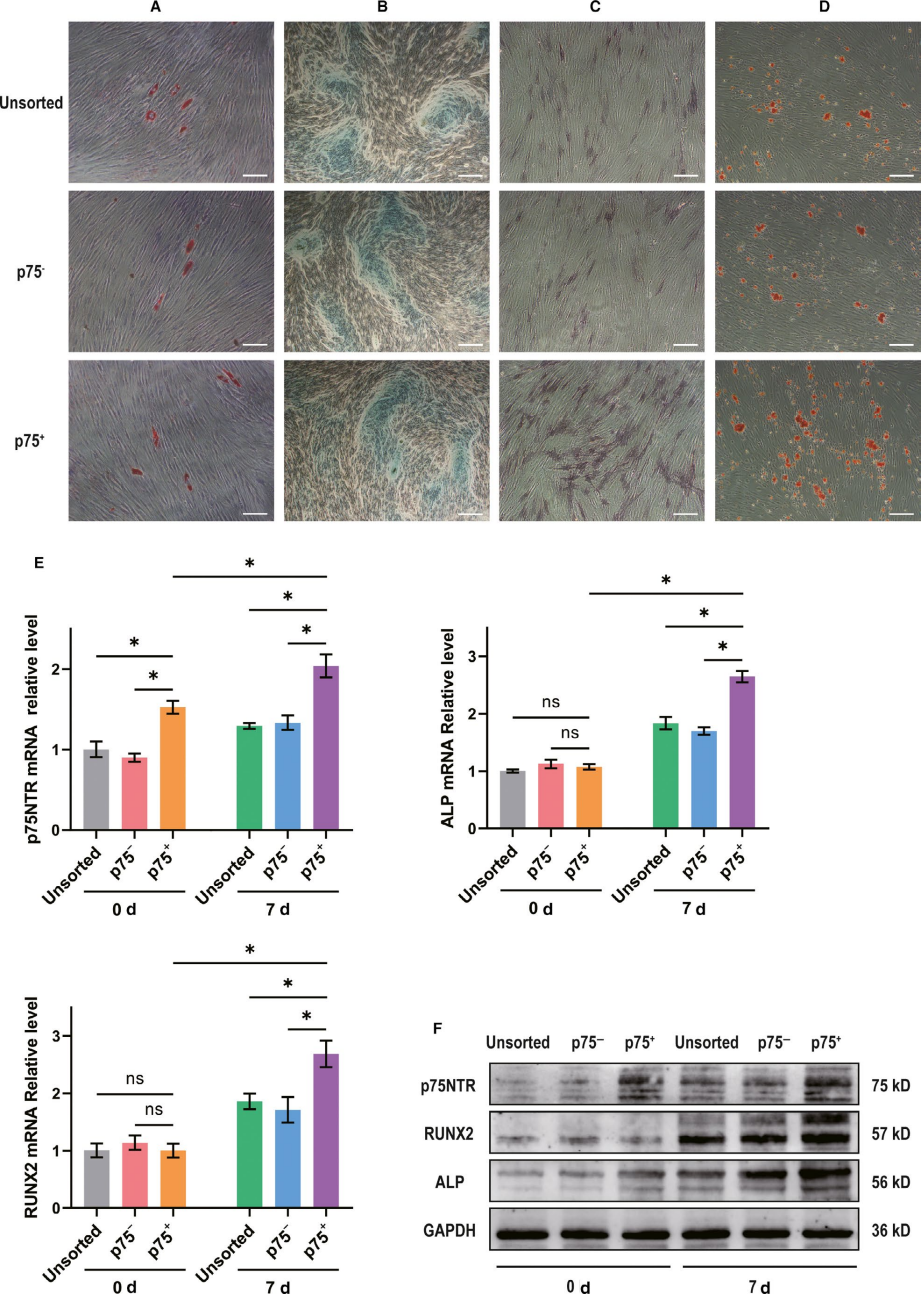
The adipogenic, chondrogenic and osteogenic differentiation among p75NTR^+^, p75NTR^−^ and unsorted hPDLSCs. (A‐B) p75NTR^+^, p75NTR^−^ and unsorted hPDLSCs were treated with adipogenic or chondrogenic induction medium for 21 days. (A) The lipids were photographed after Oil Red O staining; scale bar = 50 μm. (B) The Alcian blue staining intensity was observed by optical microscopy; scale bar = 200 μm. (C–F) p75NTR^+^, p75NTR^−^ and unsorted hPDLSCs were treated with osteogenic induction medium for 7 days or 21 days. (C) On day 7, the ALP staining intensity was observed by optical microscopy; scale bar = 100 μm. (D) On day 21, the mineralized nodules were photographed after Alizarin Red staining; scale bar = 100 μm. (E, F) On day 0 and day 7, the (E) mRNA and (F) protein levels of p75NTR, ALP and RUNX2 were detected by qPCR and Western blot, respectively, using GAPDH as a control. *P < 0.05; ns, no significant difference

After 21 days of adipogenic or chondrogenic induction, the Oil Red O staining and Alcian blue staining among p75NTR^+^, p75NTR^−^ and unsorted hPDLSCs showed that all three kinds of cells have the potential to differentiate into adipogenic and chondrogenic cell lines (Figure 4A,B). After 7 days of osteogenic induction, the ALP staining intensity was stronger in p75NTR^+^ hPDLSCs than in p75NTR^−^ and unsorted hPDLSCs (Figure 4C). Moreover, the mRNA (Figure 4E) and protein (Figure 4F) levels of p75NTR, ALP and RUNX2 were increased in p75NTR^+^, p75NTR^−^ and unsorted hPDLSCs, although the mRNA and protein levels of p75NTR, ALP and RUNX2 elevated more in p75NTR^+^ hPDLSCs than in p75NTR^−^ and unsorted hPDLSCs. After 21 days of osteogenic induction, Alizarin Red staining showed more mineralized nodules in p75NTR^+^ hPDLSCs than in p75NTR^−^ and unsorted hPDLSCs (Figure 4D).

### 3.3 p75NTR^+^ hPDLSCs expressed higher ITGA1 levels than p75NTR^−^ hPDLSCs

**Figure 5:**
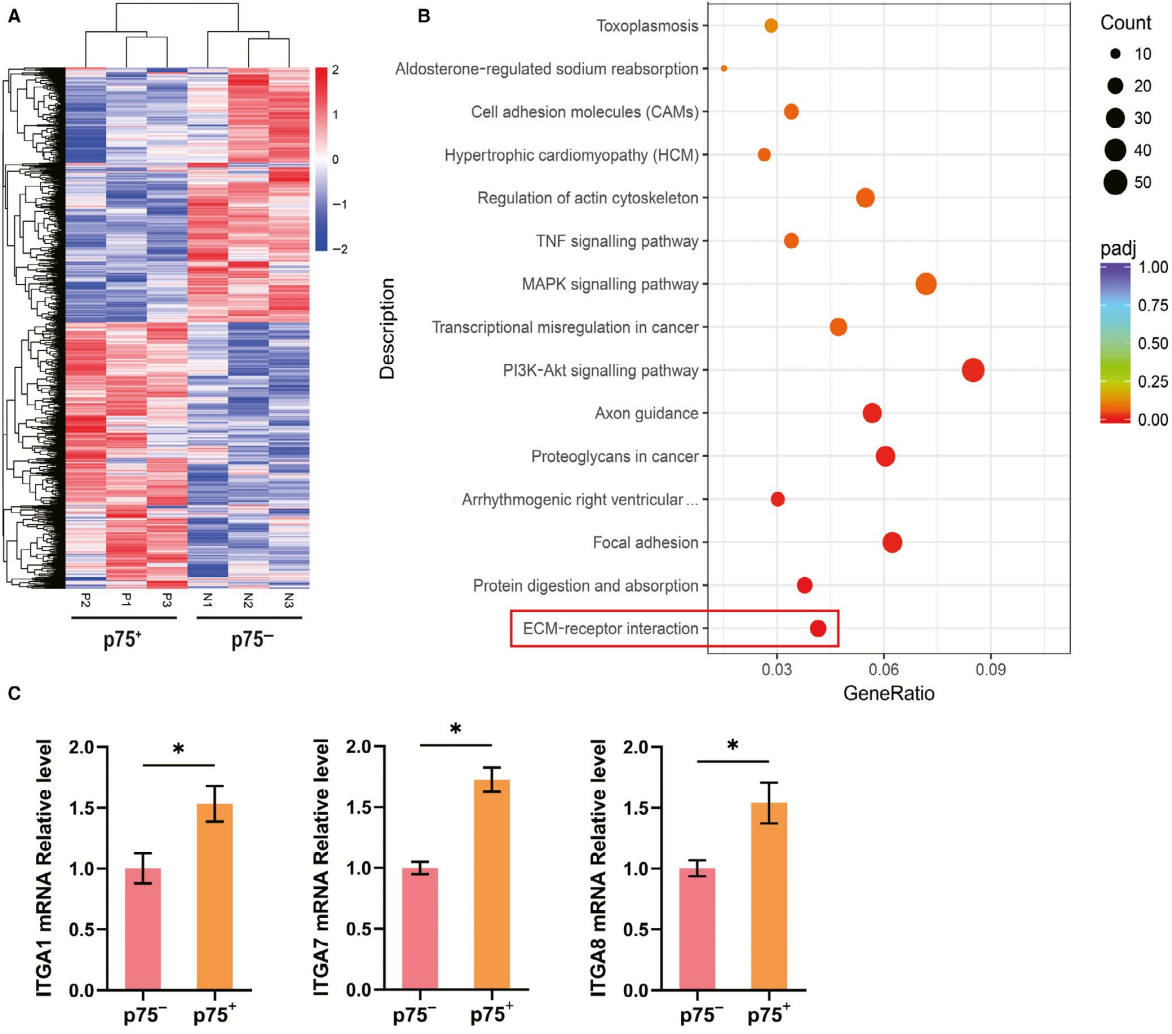
Differential expression genes between p75NTR^+^ and p75NTR^−^ hPDLSCs. (A) Heatmap of differential genes. (B) Pathway mapping of differential expression genes. (C) The mRNA levels of ITGA1, ITGA7 and ITGA7 were detected by qPCR, using GAPDH as a control. *P < 0.05; ns, no significant difference

RNA sequencing showed that 1467 genes were differentially expressed between p75NTR^+^ and p75NTR^−^ hPDLSCs in the cluster analysis (Figure 5A; Table S1). Moreover, KEGG pathway analysis showed that the differentially expressed genes were highly involved in the extracellular matrix (ECM)‐receptor interaction signalling pathway (Figure 5B; Table S2). Furthermore, qPCR confirmed that the mRNA levels of ITGA1, α7 integrin (ITGA7) and α8 integrin (ITGA8) were higher in p75NTR^+^ hPDLSCs than in p75NTR^−^ hPDLSCs (Figure 5C).

### 3.4 ITGA1 silencing inhibited osteogenic differentiation in P75NTR^+^ hPDLSCs

**Figure 6:**
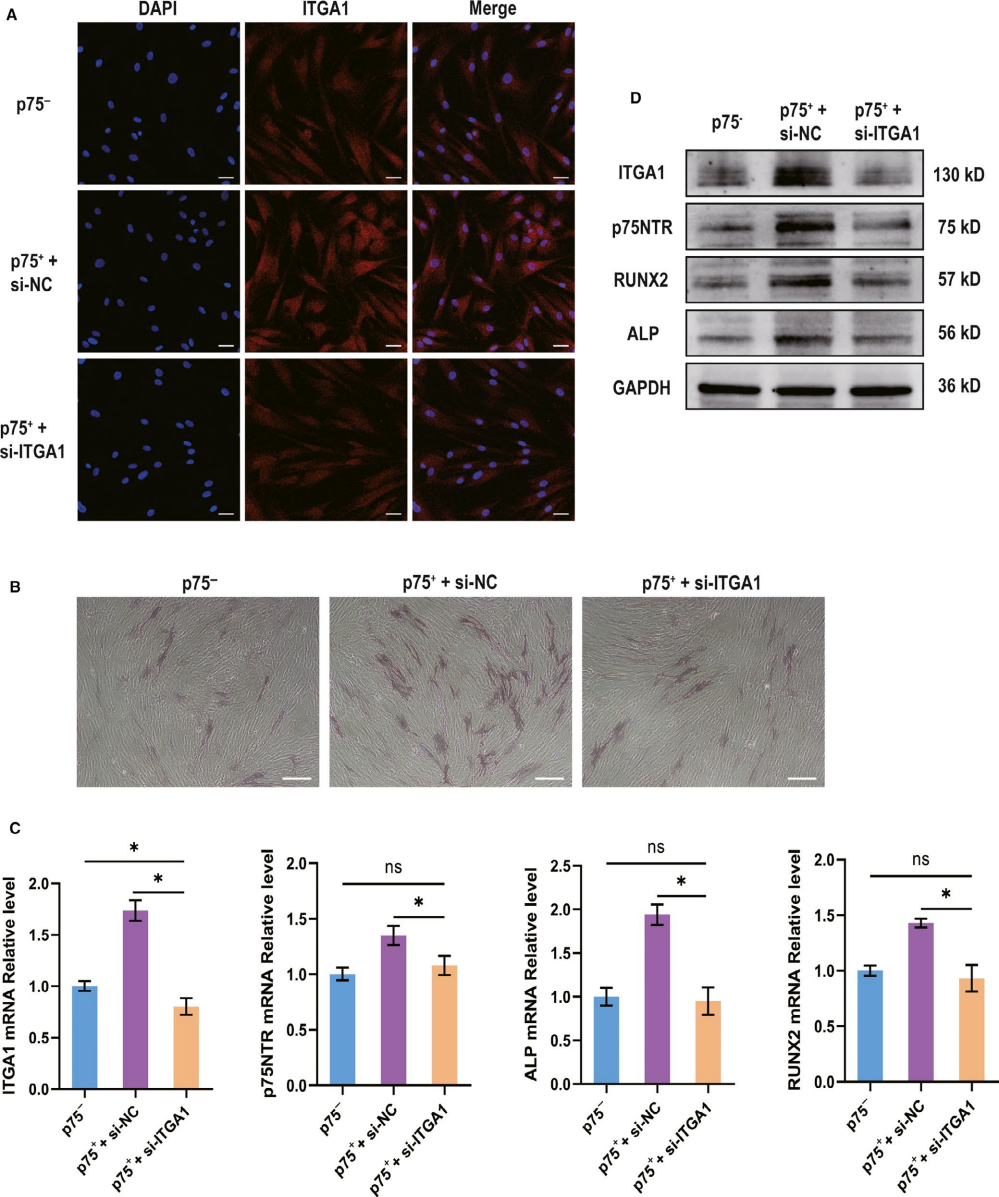
The differences in osteogenic differentiation among p75NTR^−^ hPDLSCs, p75NTR^+^ hPDLSCs transfected with negative control siRNA and p75NTR^+^ hPDLSCs transfected with ITGA1 siRNA. (A) Confocal laser scanning microscopy results of the expression of ITGA1; scale bar = 50 μm. (B–D) p75NTR^−^ hPDLSCs, p75NTR^+^ hPDLSCs transfected with negative control siRNA and p75NTR^+^ hPDLSCs transfected with ITGA1 siRNA were treated with osteogenic induction medium for 3 days. (B) On day 3, the ALP staining intensity was observed by optical microscopy; scale bar = 100 μm. (C, D) On day 3, the (C) mRNA and (D) protein levels of ITGA1, p75NTR, ALP and RUNX2 were detected by qPCR and Western blot, respectively, using GAPDH as a control. *P < 0.05; ns, no significant difference; si‐NC, negative control siRNA; si‐ITGA1, ITGA1 siRNA

The confocal laser scanning microscopy assay showed that ITGA1 was weakly expressed in p75NTR^+^ hPDLSCs transfected with ITGA1 siRNA, whereas it was significantly enhanced in p75NTR^+^ hPDLSCs transfected with NC siRNA (Figure 6A). After 3 days of osteogenic induction, the mRNA (Figure 6C) and protein (Figure 6D) levels of ITGA1, p75NTR, ALP and RUNX2 were found to be lower in p75NTR^+^ hPDLSCs transfected with ITGA1 siRNA than in p75NTR^+^ hPDLSCs transfected with NC siRNA. Moreover, the ALP staining intensity was weaker in p75NTR^+^ hPDLSCs transfected with ITGA1 siRNA than in p75NTR^+^ hPDLSCs transfected with NC siRNA (Figure 6B).

### 3.5 ITGA1 overexpression facilitated osteogenic differentiation in p75NTR^−^ hPDLSCs

**Figure 7:**
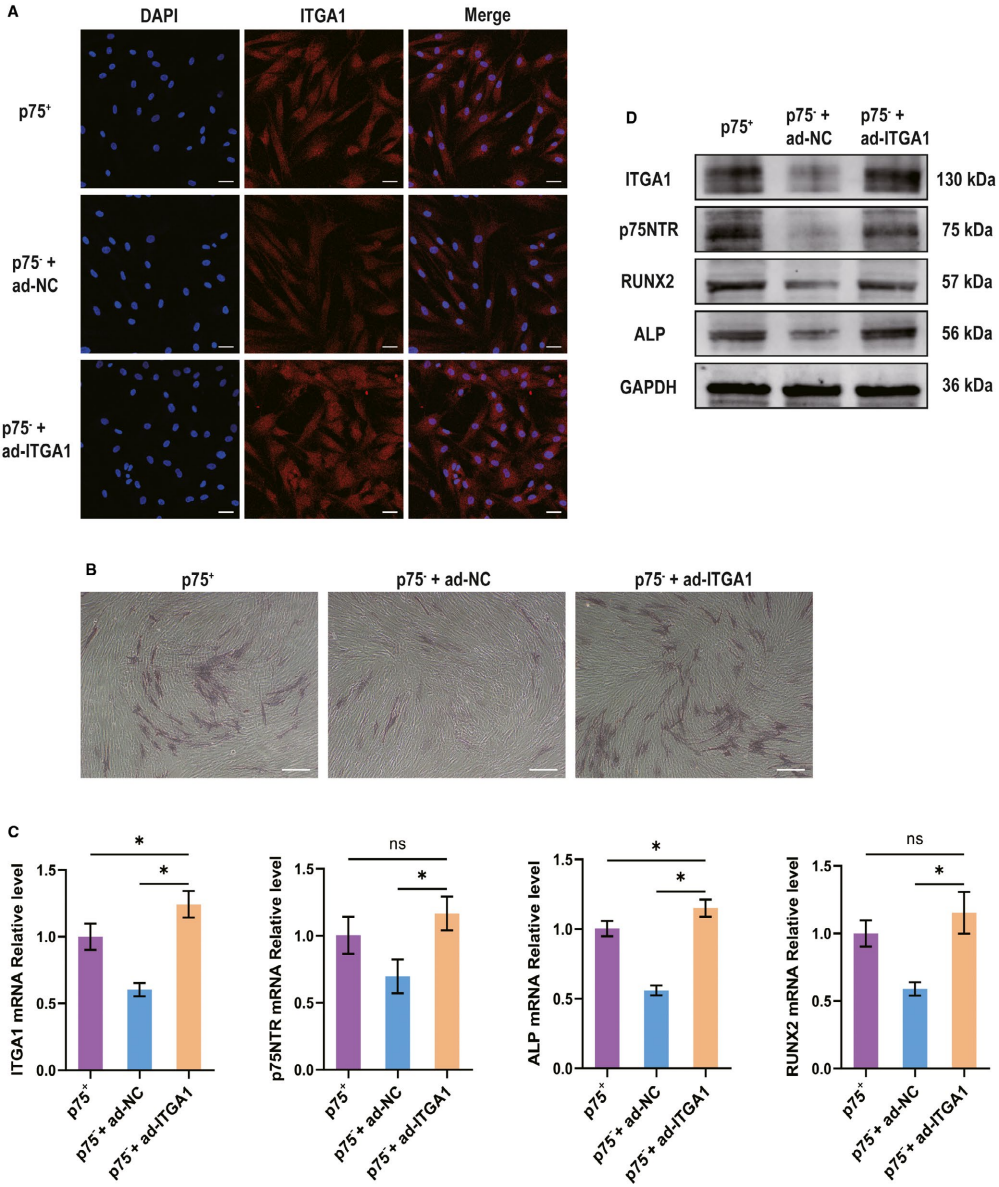
The differences in osteogenic differentiation among p75NTR^+^ hPDLSCs, p75NTR^−^ hPDLSCs transfected with negative control–overexpressing adenovirus and p75NTR^−^ hPDLSCs transfected with ITGA1‐overexpressing adenovirus. (A) Confocal laser scanning microscopy results of the expression of ITGA1; scale bar = 50 μm. (B–D) p75NTR^+^ hPDLSCs, p75NTR^−^ hPDLSCs transfected with negative control–overexpressing adenovirus and p75NTR^−^ hPDLSCs transfected with ITGA1‐overexpressing adenovirus were treated with osteogenic induction medium for 3 days. (B) On day 3, the ALP staining intensity was observed by optical microscopy; scale bar = 100 μm. (C, D) On day 3, the (C) mRNA and (D) protein levels of ITGA1, p75NTR, ALP and RUNX2 were detected by qPCR and Western blot, respectively, using GAPDH as a control. *P < 0.05; ns, no significant difference; ad‐NC, negative control–overexpressing adenovirus; ad‐ITGA1, ITGA1‐overexpressing adenovirus

The confocal laser scanning microscopy assay showed that ITGA1 expression was significantly enhanced in p75NTR^−^ hPDLSCs transfected with ITGA1‐overexpressing adenovirus, whereas it was weakly expressed in p75NTR^−^ hPDLSCs transfected with NC‐overexpressing adenovirus (Figure 7A). After 3 days of osteogenic induction, the mRNA (Figure 7C) and protein (Figure 7D) levels of ITGA1, p75NTR, ALP and RUNX2 were higher in p75NTR^−^ hPDLSCs transfected with ITGA1‐overexpressing adenovirus than in p75NTR^−^ hPDLSCs transfected with NC‐overexpressing adenovirus. Moreover, the ALP staining intensity was stronger in p75NTR^−^ hPDLSCs transfected with ITGA1‐overexpressing adenovirus than in p75NTR^−^ hPDLSCs transfected with NC‐overexpressing adenovirus (Figure 7B).

## 4 DISCUSSION

The biological role of p75NTR is controversial because it may trigger multiple cellular responses, such as proliferation, migration, apoptosis and differentiation.^22^, ^23^, ^24^, ^25^, ^26^ While most investigations have focused on the role of p75NTR in the nervous system,^15^, ^27^, ^28^ its effect on osteogenic differentiation has rarely been discussed. Prior to our study, Akiyama et al reported that p75NTR overexpression induced ALP activity and the mRNA expression of osteoblast‐related genes, including osterix and bone sialoprotein, thus promoting osteoblast differentiation in the human MG63 osteoblast cell line.^14^ Moreover, Alexander et al found that p75NTR is a differentiation marker useful for distinguishing between mineralizing jaw periosteum‐derived cells and non‐mineralizing jaw periosteum‐derived cells during the first phase of osteogenesis and can be considered an early surface marker of in vitro osteogenic capacity.^29^ In this study, we successfully isolated p75NTR^+^ hPDLSCs by fluorescence‐activated cell sorting, indicating that p75NTR can be used as a cell surface marker to isolate CNC‐derived hPDLSCs. In addition, the proportion of p75NTR^+^ hPDLSCs was as low as only 0.92%. This low percentage is consistent with the results of bone mesenchymal stem cells, of which less than 1% are p75NTR^+^.^30^, ^31^, ^32^ We subsequently induced adipogenesis, chondrogenesis and osteogenesis in P75NTR^+^, p75NTR^−^ and unsorted hPDLSCs in vitro and observed differences in their differentiation capacities. The results suggested that all three kinds of cells have the potential to differentiate into adipogenic, chondrogenic and osteogenic cell lines. Furthermore, p75NTR^+^ hPDLSCs, as CNC‐derived stem cells, possessed osteogenic potential superior to that of p75NTR^−^ and unsorted hPDLSCs given their stronger ALP staining intensity, greater mineralized node formation and higher ALP and RUNX2 expression, while there was no significant difference between p75NTR^−^ and unsorted hPDLSCs. These findings are in agreement with those of previous studies in which p75NTR^+^ dental mesenchymal stem cells were demonstrated to have the greatest osteogenic potential among the cells examined, with strong induction of osteogenic markers such as RUNX2, distal‐less homeobox 5 and bone gamma‐carboxyglutamate protein.^33^, ^34^ To explore the underlying mechanism, we further performed RNA sequencing on p75NTR^+^ and p75NTR^−^ hPDLSCs and analysed the differential gene expression profiles. The results revealed that the ECM‐receptor interaction signalling pathway might be closely related to the difference in osteogenic differentiation between these two types of cells.

The ECM is an ordered three‐dimensional network of many large molecules, mainly consisting of proteins, polysaccharides and proteoglycans, that are synthesized by cells and secreted into the extracellular space.^35^ Specific interactions between the ECM and cells are mediated by transmembrane molecules, primarily integrins and perhaps CD36, proteoglycans or other cell surface–associated components.^36^ Integrins, which contain non‐covalently bound α‐ and β‐subunits, are a family of glycosylated heterodimeric transmembrane adhesion receptors.^37^ Engagement of integrins with ECM ligands triggers integrin clustering, which activates a number of intracellular signalling pathways to regulate cytoskeletal and ECM assembly and cell biological behaviour.^38^ Accumulating evidence has shown that a crossover effect between integrins and ECM proteins is important in mediating cell proliferation and differentiation.^39^, ^40^, ^41^ On the inside of the cell membrane, integrins may regulate the osteogenic differentiation of MSCs by activating multiple signalling pathways. Marie et al demonstrated that ligand binding to integrin receptors in the ECM can promote the expression of RUNX2 through the MAPK signalling pathway.^42^, ^43^ Olivares et al reported that calcium‐dependent Wnt5a can increase the mRNA and protein expression levels of integrin and further regulate the differentiation of MSCs.^44^ Baker et al showed that the integrin‐pi3k‐akt signalling pathway plays an important role in the osteogenic differentiation of bone marrow stem cells in mice.^45^

In the present study, the differential gene expression profiles between p75NTR^+^ and P75NTR^−^ hPDLSCs revealed that ITGA7, ITGA8 and ITGA1 were the three integrins with the greatest differences in expression. ITGA7 functions as a receptor for the basement membrane protein laminin.^46^ ITGA8 regulates the recruitment of mesenchymal cells into epithelial structures, mediates cell‐cell interactions and regulates neurite outgrowth of sensory and motor neurons.^47^ ITGA1 heterodimerizes with the β1 subunit to form a cell surface receptor for collagen and laminin.^48^ Previous studies have confirmed that both collagen and laminin are closely related to osteogenesis.^49^, ^50^ In addition, Ozeki et al demonstrated that enhancement of ITGA1 expression leads to the differentiation of human skeletal muscle stem cells into odontoblasts, as demonstrated by the up‐regulation of dentin sialophosphoprotein, dentin sialoprotein and ALP.^51^ Tang et al reported that ITGA1 appears to play a significant role in the process by which iMatrix‐411, an integrin‐binding fragment derived from laminin‐411, promotes the proliferation and differentiation of odontoblast‐like cells.^52^ Therefore, we speculated that ITGA1, as a key target in the ECM‐receptor interaction signalling pathway, is a critical factor responsible for the osteogenic differentiation of hPDLSCs, and we further regulated ITGA1 expression to verify its function. The results showed that p75NTR^+^ hPDLSCs had higher expression of ITGA1 than p75NTR^−^ hPDLSCs. Moreover, ITGA1 silencing significantly inhibited the osteogenic differentiation of p75NTR^+^ hPDLSCs with weaker ALP staining depth and down‐regulated ALP and RUNX2 expression, while ITGA1 overexpression significantly facilitated the osteogenic differentiation of p75NTR^−^ hPDLSCs with stronger ALP staining intensity and up‐regulated ALP and RUNX2 expression. Moreover, after ITGA1 was regulated, the expression of p75NTR was in accordance with the changes in ITGA1 expression. Taken together, our study first demonstrated that ITGA1 promotes osteogenic differentiation of hPDLSCs under osteogenic induction conditions and that p75NTR optimizes the osteogenic potential of hPDLSCs by up‐regulating ITGA1 expression via the ECM‐receptor interaction signalling pathway. These findings not only prove p75NTR can be used to isolate homogeneous hPDLSCs with superior osteogenic potential but also provide a preliminary molecular mechanism for the effect of p75NTR on the osteogenic differentiation of hPDLSCs. In addition, as the reactions between ECM and receptors are mutual, the expression of p75NTR will also change with the regulation of ITGA1. Further studies are needed to confirm the exact mechanism of regulation between p75NTR and ECM.

In conclusion, p75NTR can be used to isolate CNC‐derived hPDLSCs. Moreover, p75NTR optimizes the osteogenic potential of hPDLSCs by up‐regulating ITGA1 expression via the ECM‐receptor interaction signalling pathway. These findings suggest that p75NTR can be used as a novel cell surface marker to identify and purify hPDLSCs, thus promoting their applications in regenerative medicine.

## CONFLICTS OF INTEREST

The authors confirm that there are no conflicts of interest.

## AUTHOR CONTRIBUTIONS

Xiujie Wen and Kun Yang conceived and designed the study; Jun Li, Manzhu Zhao, Yingying Wang, Mengjie Shen and Shuai Wang acquired the data; Jun Li, Manzhu Zhao, Mengying Tang, Meng Li and Yuting Luo analysed and interpreted the data; and Jun Li, Manzhu Zhao and Yingying Wang critically revised the manuscript.

## Supporting information

Table S1

Table S2

## Data Availability

The data that support the findings of this study are available from the corresponding author upon reasonable request.
